# Consciousness and the fallacy of misplaced objectivity

**DOI:** 10.1093/nc/niab032

**Published:** 2021-10-15

**Authors:** Francesco Ellia, Jeremiah Hendren, Matteo Grasso, Csaba Kozma, Garrett Mindt, Jonathan P. Lang, Andrew M. Haun, Larissa Albantakis, Melanie Boly, Giulio Tononi

**Affiliations:** Department of Philosophy and Communication Studies, University of Bologna, Via Zamboni, 38, 40126 Bologna, Italy; Graduate School Language & Literature, Ludwig Maximilian University of Munich, Geschwister-Scholl-Platz 1, 80539 Munich, Germany; Department of Psychiatry, University of Wisconsin-Madison, 6001 Research Park Blvd, Madison, WI 53719, USA; Department of Psychiatry, University of Wisconsin-Madison, 6001 Research Park Blvd, Madison, WI 53719, USA; Department of Psychiatry, University of Wisconsin-Madison, 6001 Research Park Blvd, Madison, WI 53719, USA; Department of Psychiatry, University of Wisconsin-Madison, 6001 Research Park Blvd, Madison, WI 53719, USA; Department of Psychiatry, University of Wisconsin-Madison, 6001 Research Park Blvd, Madison, WI 53719, USA; Department of Psychiatry, University of Wisconsin-Madison, 6001 Research Park Blvd, Madison, WI 53719, USA; Department of Psychiatry, University of Wisconsin-Madison, 6001 Research Park Blvd, Madison, WI 53719, USA; Department of Neurology, University of Wisconsin-Madison, Madison, WI 53705, USA; Department of Psychiatry, University of Wisconsin-Madison, 6001 Research Park Blvd, Madison, WI 53719, USA

**Keywords:** consciousness, functionalism, contents of consciousness, integrated information theory, space

## Abstract

Objective correlates—behavioral, functional, and neural—provide essential tools for the scientific study of consciousness. But reliance on these correlates should not lead to the ‘fallacy of misplaced objectivity’: the assumption that only objective properties should and can be accounted for objectively through science. Instead, what needs to be explained scientifically is what experience is intrinsically—its subjective properties—not just what we can do with it extrinsically. And it must be explained; otherwise the way experience feels would turn out to be magical rather than physical. We argue that it is possible to account for subjective properties objectively once we move beyond cognitive functions and realize what experience is and how it is structured. Drawing on integrated information theory, we show how an objective science of the subjective can account, in strictly physical terms, for both the essential properties of every experience and the specific properties that make particular experiences feel the way they do.

**Highlights**

- To explain consciousness scientifically, studying its behavioral, functional, and neural correlates will not suffice.
- Although explanations of consciousness must be objective, the thing to be explained is its subjective structure.
- To explain consciousness, we should account for its phenomenal structure in physical, causal terms.
- Phenomenal space—the feeling of extendedness—provides an initial opportunity to develop such an account.
- An overall good explanation of consciousness must draw on introspection and reason along with empirical correlates.

One learns early on that science consists in objectively explaining objective properties. This notion has been a cornerstone of the scientific method since Galilei, who purposefully set aside subjective properties—the way things feel to a subject—as outside the purview of science. It has proven extraordinarily successful to focus on objective properties—those that can be defined operationally through observations and perturbations. It has been so successful, in fact, that scientists have begun to investigate the objective correlates of consciousness. Yet if we wish to understand consciousness, what need to be explained objectively are its subjective properties. Can this be done through science?

In what follows, we first briefly outline standard scientific approaches to objective correlates of consciousness—namely, behavioral, functional, and neural correlates. We then consider the recurring claim that only functional correlates of consciousness can be accounted for by science, or even that they may be all there is to explain. This claim, we argue, commits the “fallacy of misplaced objectivity.”^1^ Subjective properties of experience can and should be studied objectively. This becomes possible if we characterize what consciousness “is”, not just what it “does.” Drawing on integrated information theory (IIT), we illustrate how both the essential properties of every experience and the specific properties of particular experiences can be accounted for objectively and tested empirically.

## The objective study of objective correlates of consciousness

Since the nineteenth century, researchers interested in the mind have tried to employ methods and tools that would ensure objectivity, varying primarily in what they considered as the proper standards of science. For example, Fechner and the early psychophysicists and psychologists considered “laws” as the paradigm of scientific objectivity and sought to establish laws connecting the mental and the physical (Wundt 1904; Fechner 1948; Pigorini *et al.* 2015). In the twentieth century, the emphasis shifted to characterizing behaviors, understanding cognitive functions irrespective of their implementation, or identifying the precise neural correlates of consciousness and its contents. Each of these research programs has contributed extensively to the scientific study of consciousness.

### Behavioral correlates

John Watson, the figurehead of behaviorism, admitted the existence of consciousness but claimed that it had “never been seen, touched, smelled, tasted, or moved” (Watson and McDougall 1929). A truly scientific psychology should only consider observable stimuli and responses, which could be characterized objectively, treating everything in between as a black box. The way things feel, thought Watson, lay outside the scope of objective scientific explanation.^2^

At the bedside, neurologists still use behavioral observations and carefully designed stimulus–response tests to assess the presence or absence of consciousness, although such tests have clear limitations. For example, the commonly used Glasgow Coma Scale considers patients’ ability to move their eyes, speak, and move their body (Posner *et al.* 2019). On the other hand, we now think that some behaviorally unresponsive patients are conscious because, like healthy subjects, they can activate the appropriate cortical regions when asked to imagine different scenarios (Owen *et al.* 2006). Conversely, behaviors such as tracking a target with the eyes may occur in the absence of experience (Bruno *et al.* 2010). In the course of general anesthesia, too, consciousness is regularly assessed based on the loss of behavioral responsiveness. However, some anesthetized patients can respond to commands if an arm is left unparalyzed (Sanders *et al.* 2012; Linassi *et al.* 2018) or report vivid dreams after surgical-level ketamine infusions (Sarasso *et al.* 2015). This should come as no surprise since during natural sleep we remain largely immobile and unresponsive, even though we dream through much of the night (Siclari *et al.* 2017). The limitations of behavioral correlates are clearest in assessing the presence of consciousness in animal species very different from us or in intelligent robots. In fact, a properly equipped robot could easily pass a neurological examination much better than many conscious patients in a neurology ward. Should we infer that the robot is more conscious than they are?

### Functional correlates

Starting in the 1950s, researchers in computer science, psychology, and linguistics realized that treating the mind (and consciousness) as a black box was hopelessly inadequate. If we wish to explain how the mind works, they thought, we must consider not just inputs and outputs, but also internal states and operations on them. Science had to open the black box and replace it with multiple interacting boxes, corresponding to various cognitive functions.

The cognitive sciences have been remarkably successful, especially when married to neuroscience (Gazzaniga *et al.* 2019; Postle 2020). We have learned a great amount about cognitive functions such as attention, working memory, executive functions, and language. Broadly speaking, however, the objectives of study of the cognitive sciences are the functions and objective manifestations of the mind, rather than consciousness itself. For many decades, if consciousness was mentioned at all, it was identified with the content of attention and working memory, which could be cognitively manipulated and reported.^3^

Lately, it has become acceptable to refer to consciousness explicitly. However, the understanding is that only what is cognitively “accessible” and thus reportable can be the object of science, in contrast to the purely “phenomenal” properties of consciousness—what experience is and how it feels. And yet, it is precisely those phenomenal aspects that make consciousness what it is and that we should hope to explain. Who would settle for going through life carrying out every function normally, including access and report, but without phenomenology—doing everything while experiencing nothing? Similarly, we need to know whether an unresponsive patient feels anything, not just whether she can access those feelings or not. And we would want to know whether an intelligent robot—one that can behave autonomously, attend, remember, and report—is having experiences or merely acting as if it did.

### Neural correlates

In the 1990s, neuroscientists started approaching consciousness in earnest and programmatically. Crick and Koch (1990) promoted the search for the neural correlates of consciousness (NCC), arguing that “the problem of consciousness can, in the long run, be solved only by explanations at the neural level.” The full NCC can be defined as the minimum neuronal mechanisms jointly sufficient for any one specific conscious experience (Chalmers 2000). Crick and Koch (2003) proposed several candidates, such as neurons firing at around 40 Hz or neurons projecting directly to prefrontal cortex.

A useful distinction is between content-specific NCC—the neural mechanisms specifying particular phenomenal contents, such as a particular color, sound, face, or place—and the full NCC—the union of the content-specific NCC that contribute a particular experience (Koch *et al.* 2016). Content-specific NCC can be investigated through a combination of recording, stimulation, and lesion data, revealing which neural mechanisms contribute to experience. The full NCC can be identified by comparing conditions in which consciousness as a whole is present or absent—e.g. by comparing brain activity during dreaming and dreamless sleep (Siclari *et al.* 2017). It is also important to distinguish between NCC and background conditions for consciousness. For example, just like blood flow to the brain, an adequate level of activity of arousal systems in the reticular formation of the brainstem is typically required for being conscious, but this does not mean that brainstem cell groups contribute specific contents to experience. The goal of NCC research is to gather enough empirical evidence to establish objectively which brain areas and neural elements can or cannot support consciousness and its contents—e.g. the cerebral cortex and/or thalamus, the front and/or back of the cortex, some particular cell layers or types, some particular modes of firing, and so on (Koch *et al.* 2016).

NCC research has opened the doors to powerful new tools that allow us to decode the contents of experience from distributed neural activities in the cerebral cortex (Poldrack 2011; Davis and Poldrack 2013). The promise of these methods was clear early on: “Using information that was present in the multivariate pattern of responses to stimulus features, we could accurately predict, and therefore track, participants’ conscious experience from the fMRI signal alone while it underwent many spontaneous changes” (Haynes and Rees 2005). Techniques have advanced rapidly, allowing researchers to partially decode the content of dreams (Horikawa *et al.* 2013), to increase the precision of decoded contents (Kriegeskorte and Douglas 2019), and to map the cortical representations of similar and different contents (Diedrichsen and Kriegeskorte 2017).

Decoding the contents of consciousness from brain activity patterns is admittedly not the same as explaining how they feel. However, at least some neuroscientists have pursued NCC research with the hope to gain some critical insight into the subjective properties of experience (Crick and Koch 2003): “No one has produced any plausible explanation as to how the experience of the redness of red could arise from the actions of the brain. It appears fruitless to approach this problem head-on. Instead, we are attempting to find the neural correlate(s) of consciousness (NCC), in the hope that when we can explain the NCC in causal terms, this will make the problem of qualia clearer.”

## The fallacy of misplaced objectivity

But should we even hope to account for the subjective properties of consciousness? Following the Galilean tradition, many believe that all we should do—and can hope to do scientifically—is to objectively study the objective correlates of the presence and contents of experience, as accessed through cognitive functions such as attention and working memory. This view, we argue, commits the fallacy of misplaced objectivity: the assumption that only objective properties should and can be accounted for objectively through science.

### Consciousness is what consciousness does? Substituting function for phenomenology

“We argue that all theories of consciousness that are not based on functions and access are not scientific theories. […] A true scientific theory will say how functions such as attention, working memory and decision making interact and come together to form a conscious experience” (Cohen and Dennett 2011). This quote is an especially explicit assertion that all we can study scientifically is what subjects can access through attention, and eventually report, about the presence and the contents of consciousness. In this view, only functions matter since they can be studied objectively by independent observers. This attitude is widespread (e.g. Doerig *et al.* 2019; Graziano 2019; Frankish 2021) and seemingly justified by the Galilean notion of science as the objective investigation of objective properties. Anything beyond function has been considered inexistent (Churchland 1981), illusory (Frankish 2016), or irredeemably phenomenal—i.e. subjective—and thereby outside the scope of science.

Studies of change blindness and partial report, such as the Sperling task, are often brought up in support of a functionalist outlook (Cohen and Dennett 2011). In many such studies, subjects may think that they are experiencing a rich visual scene—one containing numerous objects, contours, or colored patches. But when they are rigorously tested, it turns out, to their surprise, that they failed to notice obvious changes in the scene, such as the disappearance of a prominent object, or that they can report correctly at most 3–4 letters out of a large array. Objectively, we can justifiably infer that subjects did experience what they could access and report—a few items plus some overall gist or “summary statistics.” But any further inference about what they may have experienced subjectively—“inside”—is not supported by what they can report objectively—“outside.”

In this view, therefore, the purported richness of subjective contents should be considered a confabulation or an illusion. And if it is not an illusion, subjective richness is something that cannot be investigated scientifically because there is nothing to go on but the subjects’ objective performance. In other words, from the point of view of science, consciousness is what consciousness does.

### Do I say what I see, or do I see what I say? Putting the cart of access before the horse of experience

The kind of evidence just mentioned has recently been questioned by experiments suggesting that subjects’ experiences may be richer than assumed based on standard reporting paradigms (Block 2011, 2014; Haun *et al.* 2017; Usher *et al.* 2018; Fu *et al.* 2021). Also, most of the Sperling-type evidence is based on reports of high-level categories, such as objects or characters.^4^ Here, however, we emphasize two general points.

First, treating access and report as the gold standard for judging experience puts the cart before the horse, or, more elegantly, inverts the epistemic order. Just like behaviorism, the view that only functions matter forgets that the validity of access and report is grounded in experience, not the other way around. When we are conscious, we can usually engage in various cognitive functions—we can attend to contents, keep them in memory, and manipulate them; we can introspect and reflect on what we experience; and we can usually translate some contents into words. For this reason, we justifiably take reports from other beings who look and act like us as plausible indications of what they experience. But we should remember the proper order of things: it is only because we are conscious that we can report our experiences. For this reason, reports can be treated as convenient proxies for consciousness. Put differently, “I am seeing it, so I can say it” is sensible; “I say it, so I must be seeing it” is absurd.

Second, to claim that all there is to experience is what we can do with it commits the fallacy of misplaced objectivity: it places objectivity not just where it belongs—in the way we explain things—but also where it does not belong—in the things that need to be explained. With consciousness, unlike anything else in science, what needs to be explained is precisely its intrinsic, subjective properties—those that are essential to all experiences and those that are particular to a given experience. If we fall into the fallacy and replace experience with functions, we end up with an explanation of those functions, not of experience.^5^

To be sure, subjective properties may seem impossible to explain, at least at first. They may be outright ineffable (how could I communicate in words what nausea feels like?). They may defeat our analytical skills (how would I decompose the way the face of Mona Lisa looks?). They may exceed our ability to access their content sequentially (how would I articulate how a Jackson Pollock painting looks if I had just one quick glance at it?). Yet each experience feels the particular way it does, and there must be an explanation for how it feels.

### Science or magic? Banishing experience rather than accounting for it

Indeed, there must be a reason why things feel the way they do—why space feels extended; time feels flowing; objects, colors, and sounds feel the way they do; and so on. All these subjective properties cannot just happen to feel that particular way in a gratuitous, arbitrary manner. This is one more reason why it is counterproductive to restrict scientific investigations to access and report. Far from ensuring scientific objectivity, it exiles the way experience feels to the realm of magic rather than science.

Moreover, we cannot ignore the overwhelming evidence that ties the content of experience to specific regions of the brain. Why would some regions contribute to experience and others not at all? Why would the same regions contribute to experience when we are awake but not in dreamless sleep? And why would visual cortices contribute to visual experiences “painted” on a 2D canvas, and auditory cortices to auditory experiences “streaming” in an ever-evolving present? There must be an answer to such questions.

## The objective study of subjective properties of experience

Against the fallacy of misplaced objectivity, we argue that while the explanation must be objective, what must be explained is subjective—namely, consciousness with all its properties, which is intrinsic to the subject of experience (Tononi 2015). Moreover, subjective properties can be accounted for objectively using the methods of science: we should explain them based on objective properties of the neural substrate of consciousness, rather than consign them to the realm of the inexplicable. The conclusion is that a scientific account of the way experience is and how it is structured intrinsically—rather than of what it can do and what functions it enables extrinsically—should be pursued and can be pursued.

**Figure 1.**
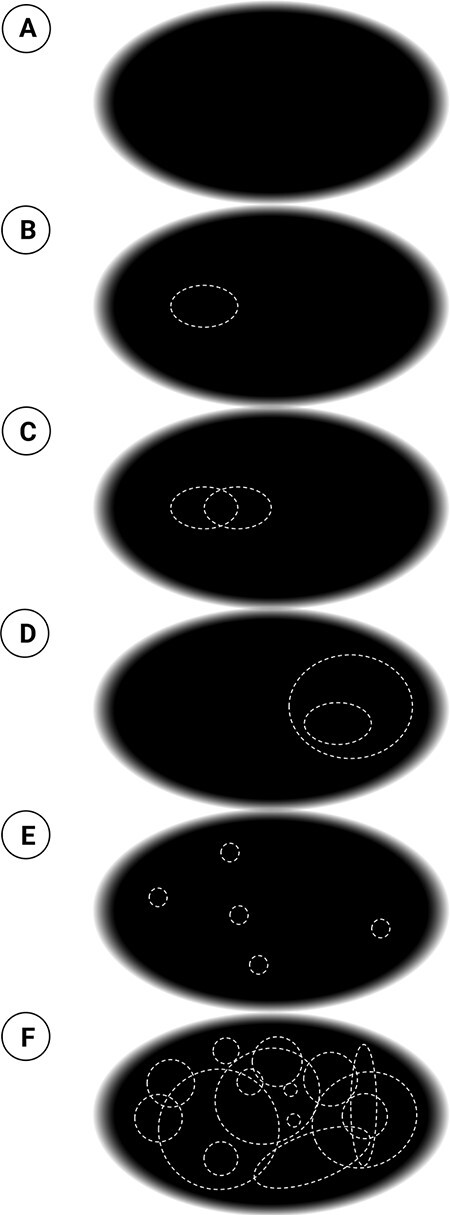
Panels A to F portray the visual experience of an observer looking at a dark screen on which dashed ovals appear. The illustration serves to guide the reader through introspecting the phenomenal structure of space, as described in the text.

As an illustration, consider the following example. Suppose you are an experimental subject looking at a large, dark screen, fixating in the middle (Fig. 1A). You are obviously having an experience, regardless of whether you tell yourself or anybody else. You are now asked by the investigators to report what you are seeing. Suppose you answer with a kind of summary statement: “I see a vast, empty canvas.” Then, slightly to the left of fixation, a bright, dashed oval is flashed (Fig. 1B). You dutifully report that you see a dashed oval and where you see it. The session goes on, and you report seeing two ovals partially overlapping to the left of fixation (Fig. 1C) and then two ovals to the right, one of which includes the other (Fig. 1D). Then you see five ovals of various sizes at seemingly random locations, although you can still tell where they appeared on the screen (Fig. 1E). Finally, tens of dashed ovals are flashed at once all over the screen (Fig. 1F). You then report seeing indeed many ovals all over the screen, but you cannot say where they were located individually, whether and how they were overlapping, or how distant they were from one another.

Based strictly on what you can report at any one time, the investigators might conclude that what you are seeing at any one time is at most a handful of ovals over a vast background.^6^ Beyond those few ovals you can report about, nothing much can be said objectively. The investigators, from their extrinsic perspective, are trying to account for what you can do with the experience you are having, not for how it feels from your intrinsic perspective. In other words, they presume what you say to be what you see and thus account for function rather than phenomenology. They may then fruitfully investigate, in neural terms, the mechanisms responsible for the capacity limitations of cognitive functions such as attention and working memory.

But if we want to understand consciousness as such, rather than cognitive functions, we should try to answer two questions: What makes any experience “an experience”? And what makes a specific experience “that experience”? To answer the first question, we should identify the “essential” properties of consciousness—those that are true of every conceivable experience, whether of a dark screen, a landscape, a musical chord, a pain, or a flavor. For instance, what does it take for your current experience—like every conceivable experience—to be a unitary whole and yet be composed of parts? To answer the second question, we should try to identify “specific” properties of particular experiences. For instance, what does it take to make your current experience feel spatially extended? What makes the ovals seem like regions of space? What makes them seem to include or overlap other ovals, or to be located at a certain distance from one another?

In other words, to understand consciousness as such, we should ask questions about its intrinsic structure, not about its extrinsic functions. Of course, the proof of the epistemic pudding is in its scientific outcome. Both the essential and specific properties of consciousness must be accounted for in neural terms. In doing so, we should make use of introspection and reasoning as well as of the behavioral, functional, and neural correlates of consciousness. But the role of these objective correlates should be clear: to validate the explanation, not to surreptitiously replace the thing to be explained.

Below we outline the approach we have taken to develop such an account, namely, IIT (Oizumi *et al.* 2014; Tononi 2015; Tononi *et al.* 2016). Our goal here is not to present IIT in any detail or to argue for its principles, validity, or uniqueness. Instead, we employ IIT to suggest how one can try to explain the subjective in objective terms—i.e. to explain phenomenal properties, both essential and specific, in terms of physical properties.

### The essential properties of every experience: accounting for the presence and absence of consciousness

**Figure 3.**
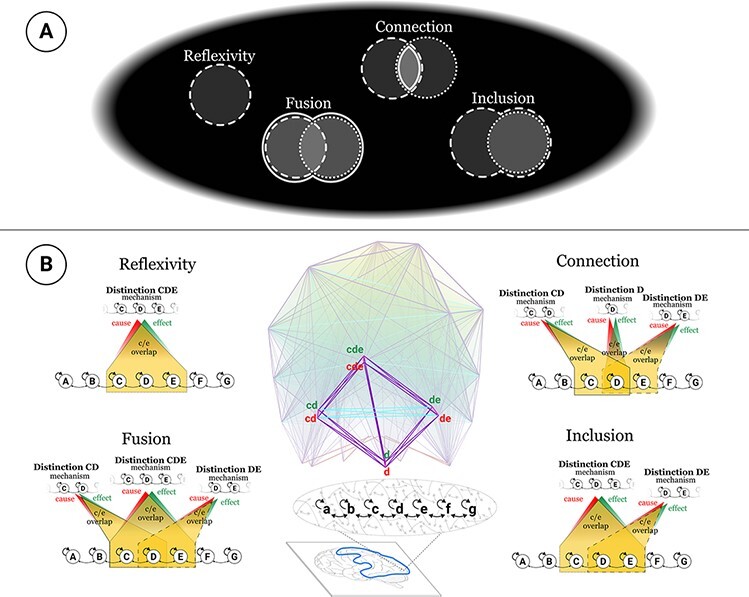
Extendedness in phenomenology and cause–effect structures. A) The phenomenal properties that characterize spatial extendedness and B) the physical properties that correspond to them. The polytope in the bottom center depicts the unfolded cause–effect structure of a seven-unit grid. The distinctions “d,” “cd,” “de,” and “cde” are singled out to illustrate the four types of causal relations that make up extendedness. Cyan edges indicate partial overlap between causes and/or effects, purple edges asymmetric full overlap, and magenta edges symmetric full overlap [see Haun and Tononi (2019)]. “Reflexivity”: each phenomenal spot overlaps itself; similarly, distinctions in the cause–effect structure fully overlap (by virtue of the overlap between their cause and effect). “Connection”: spots partially overlap other spots, and the overlap corresponds to another spot; similarly, distinctions partially overlap with their causes and effects, and the overlap corresponds to the cause and effect of other distinctions. “Fusion”: the union of two connected spots corresponds to another spot; similarly, the union of two connected distinctions is also a distinction. “Inclusion”: every spot includes and is included by other spots; similarly, every distinction includes and is included by other distinctions. For a more detailed analysis, see a companion paper (Grasso *et al.* 2021).

According to IIT, there are five essential properties that are true of every conceivable experience, even the experience of seeing a featureless, “empty,” dark screen that occupies one’s entire field of vision (like in Fig. 1A):

1. Every experience is “subjective”—intrinsic to a subject. It is for the subject of experience, from the subject’s own intrinsic perspective, rather than for something extrinsic to that subject. Thus, the vast, empty canvas is experienced by me, not by somebody else.
2. It is “structured,” being composed of phenomenal distinctions and relations among them. A phenomenal distinction is any “part” of an experience, no matter how salient or subtle. In the case of the empty canvas, distinctions are “spots”—patches of the canvas, of any size or shape and at any location. Figure 1B–F highlights but a handful of spots as dashed ovals. However, countless spots are present in any spatial experience whether they are highlighted as in the figure or not.^7^ Relations between these spots can be understood as the way the spots connect to, fuse with, and include one another (see Fig. 3A).
3. Every experience is “specific”—the particular way it is—rather than generic. For example, the canvas may be empty, or it may contain a few dashed white ovals (as in Fig. 1) or other shapes, at any conceivable location, which may overlap with each other in every conceivable way. And, of course, the canvas could be black or white, blue or red, or it could be any of a countless number of specific scenes.
4. Every experience is also “unitary.” Thus, the canvas I see cannot be reduced to a left side and a right side that are experienced independently—if it were so, there would be two independent consciousnesses, rather than one. In Fig. 1E, e.g. my experience is of all five ovals as a unity, despite their separation and regardless of my point of focus.
5. Finally, every experience is “definite”—it has borders, containing what it contains, neither less nor more. It does not contain “less” than it contains—e.g. it does not contain only the spots on the left side but not those on the right side, or only the spots in the periphery but not those in the center. It also does not contain “more” than it contains—e.g. spots behind my head.

In summary, my experience of the canvas—like every experience—is an intrinsic, “phenomenal structure” that is specific, unitary, and definite. Different experiences are different phenomenal structures, depending on the particular distinctions and relations that compose them. The structure of experienced space is characterized by extendedness, that of experienced time by flow, that of experienced objects by a binding of general concepts with particular features, and so on. It should be clear, then, that an experience is an intrinsic structure, not a function. It is characterized by what it is, not by what it does.

The intrinsic, phenomenal structure of an experience should have a physical explanation; otherwise how an experience feels would be consigned to magic rather than science. “Physical” is understood in an operational sense: something is physical if it can “make a difference” and “take a difference”—if it has cause–effect power, as demonstrated through observations and manipulations (Tononi 2015). On this basis, IIT proposes an explanatory identity: an experience—a specific “phenomenal structure”—is identical to a specific “cause–effect structure.”

A cause–effect structure captures the causal powers of a physical substrate in a particular state—say, a network of neurons, some active and some inactive. To obtain a cause–effect structure, we must “unfold” the causal powers of the substrate in full.^8^ This means systematically observing and manipulating the substrate to assess precisely how subsets of neurons make a difference to and take a difference from other neurons of the substrate. Doing so yields a set of causal distinctions and relations that allow us to evaluate whether the causal powers of the unfolded substrate of an experience can account for its essential phenomenal properties—being intrinsic, structured, specific, unitary, and definite.

**Figure 2.**
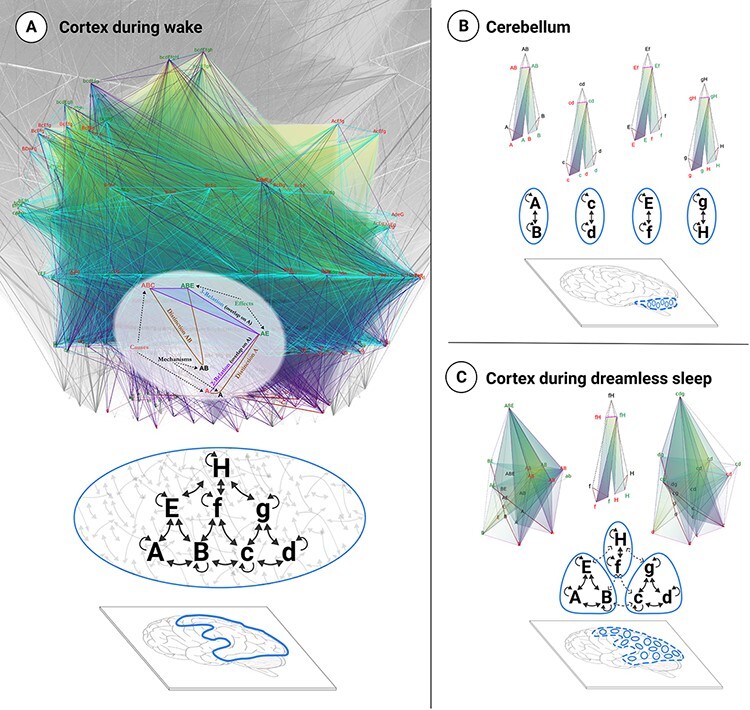
Unfolded cause–effect structures for three “brain-like” physical substrates. A) A highly integrated eight-unit network in state ABcdEfgH (where uppercase indicates ON and lowercase OFF) unfolds into a rich cause–effect structure with many distinctions and relations. The cause–effect structure of this eight-unit network is meant to illustrate a small part of a much larger cause–effect structure (grayed out in background) that is presumably specified by the physical substrate of human consciousness. The overlay highlights how a distinction (brown) specified by a mechanism (black) comprises a cause (red) and/or an effect (green). Relations among causes and/or effects (overlaps among them) are shown as lines, faces, and volumes, with colors denoting different types of relations. The figure indicates that during wakefulness, the physical substrate of consciousness—constituted of a large number of neurons arranged as “pyramids of grids” and located predominantly in posterior cortical areas (outlined in blue)—can specify a cause–effect structure with high *Φ* (Tononi *et al.* 2016). B) When eight units are connected pairwise as parallel modules with minimal intermodular connectivity, the network specifies four separate, minimal cause–effect structures, each with very low *Φ*. Modular connectivities are found, for instance, in the cerebellum. C) Even when units are interconnected as in A, their causal interactions can be disrupted by changes in neuromodulation and excitability (dashed connections) that lead to a breakdown of causal interactions, severely reducing the network’s capacity for integrated information. As a consequence, the single, rich cause–effect structure of high *Φ* (Panel A) “disintegrates” into multiple disjoint ones, each with low *Φ* (Panel C). According to IIT, this breakdown in the capacity for integrated information, consistent with the breakdown of perturbational complexity, accounts for the loss of consciousness during deep sleep early in the night (Massimini *et al.* 2005; Pigorini *et al.* 2015). While the purpose of this figure is purely illustrative, the cause–effect structures shown were obtained by computationally unfolding the associated networks of units in their particular state, obtaining their irreducible causal mechanisms with their causes, effects, and relations [for details, see Haun and Tononi (2019)].

Note that by “structure”—phenomenal or physical—we mean an actual entity, existing “here and now” and composed of distinguishable parts (distinctions) related among themselves in various ways (relations). As a structure, a substrate with all its causal powers unfolded is the physical correspondent of an experience—an entity or “thing” whose properties are actual, rather than a “process” that performs some “function” over time. Figure 2A depicts the cause–effect structure unfolded from a simple eight-neuron network. All causal distinctions are represented (as vertices), but only a small fraction of the causal relations among them are shown (as edges, faces, and volumes).

The identity between an experience and a cause–effect structure is “explanatory” in the sense that every aspect of a particular phenomenal structure should be accounted for by the specific distinctions and relations of its corresponding cause–effect structure.^9^ A sketch of such an account is given in the next section for the feeling of spatial extendedness. Moreover, using the measure of integrated information, or *Φ*, one can quantify the extent to which the unfolded physical substrate exists as a single, irreducible entity that satisfies the essential properties of consciousness.^10^

Much of the initial work in IIT has focused on two straightforward predictions: *Φ* should be high for the brain network that constitutes the physical substrate of consciousness (roughly speaking, the full NCC) and absent (or negligibly low) for neural networks that do not support consciousness (Fig. 2A vs. B). Moreover, the same physical substrate should satisfy the five essential properties when we are conscious and cease to do so when we lose consciousness (Fig. 2A vs. C).

In practice, we test these predictions using empirical approximations inspired by *Φ*. For example, the perturbational complexity index (Casali *et al.* 2013) coarsely quantifies how much a neural substrate is both differentiated and integrated. To be differentiated means having a large repertoire of possible states and is a prerequisite for being a structured and specific entity. To be integrated means behaving causally as a single entity, which is a reflection of being unitary and definite.

These predictions have been examined both experimentally and with computer models of brain architectures (Balduzzi and Tononi 2008; Tononi *et al.* 2016). The results so far indicate that neural substrates that support human consciousness—especially posterior cortical areas organized as “pyramids of grids”—have an anatomical connectivity appropriate for high *Φ*. By contrast, neural substrates that do not support consciousness, such as the cerebellum, have the wrong anatomy. Even though the cerebellum has five times more neurons than the cerebral cortex and is connected to sensory inputs, motor outputs, and indirectly to the cortex itself, its architecture is primarily feed-forward and modular, which rules out high values of *Φ* (Fig. 2A vs. B). Moreover, studies in healthy subjects during wakefulness, dreamless and dreaming sleep, and general anesthesia indicate that the loss and recovery of consciousness are associated with the breakdown and recovery of the capacity for information integration in the corticothalamic system (Casarotto *et al.* 2016; Tononi *et al.* 2016) (Fig. 2A vs. A).^11^

### The specific properties of particular experiences: accounting for the way space feels

What about the specific properties that make every experience feel the particular way it does?—or at least that make it feel like an experience of a particular kind? Consider again the example above, of seeing a vast, empty canvas. The experience, like any other, is subjective, structured, specific, unitary, and definite. It also feels the specific way it does—namely, “spatially extended” as opposed to, for instance, “flowing in time.”^12^In fact, most experiences involving both visual and body space feel extended. Can we account for the way these kinds of experiences feel in physical terms?^13^

In this case, IIT begins again by identifying the fundamental phenomenal properties that make space feel the way it does—those that are true of every conceivable spatial experience (Haun and Tononi 2019). As introduced above, every spatial experience is composed of phenomenal distinctions that we call “spots.” In fact, the entire canvas of phenomenal space comprises countless spots of every shape and size, which are bound together by four types of phenomenal relations: “reflexivity” (every spot overlaps itself), “connection” (every spot partially overlaps some other spots), “fusion” (every spot is a fusion of connected spots and fuses with other connected spots), and “inclusion” (every spot includes and is included by other spots) (Fig. 3A). Other properties of spatial experience can be characterized as phenomenal “substructures” composed of spots and their relations. These include “regions” of space, “locations” in space, “sizes” and “boundaries” of regions, “distances” between spots, and so on (Haun and Tononi 2019).^14^

Assuming these properties do indeed capture what is specific about phenomenal extendedness, we should then try to provide a scientific account of each of them in physical terms. According to IIT, this means that we should identify a neural substrate whose unfolded causal powers—its cause–effect structure—correspond one-to-one to the phenomenal structure of spatial experiences.

As described in detail elsewhere (Haun and Tononi 2019), the phenomenal properties in question can be fully accounted for physically by the cause–effect structure unfolded from 2D grids constituted of neurons connected locally by lateral connections. That is, neurons arranged in grids display systematic patterns of cause and effect that can account for the phenomenal relations of extendedness (reflexivity, connection, fusion, and inclusion, Fig. 3B), while other substrates, such as networks of randomly connected neurons, cannot. Moreover, the cause–effect structure unfolded from a grid-like substrate satisfies these properties irrespective of whether units are active or not, as long as the units are ready to be turned on or off.

This account of spatial experience is supported by ample empirical evidence. For example, lesions in visual cortical areas produce not just extrinsic blindness, in the sense that patients do not detect objects in some portions of their visual field, but also an intrinsic loss of experienced space: subjectively, a region of visual space ceases to exist—it is simply “not there” (Pollen 1999; Hazelton *et al.* 2019). After a severe stroke, resection of one occipital lobe, or callosal disconnection, the contralateral half of experienced space disappears in both perception and imagination (Bisiach and Luzzatti 1978; Farah *et al.* 1992; Butter *et al.* 1997). In fact, because contralateral space does not exist subjectively, patients are not aware that anything is amiss and may only realize indirectly that something is not right (Sperry 1968; Gilhotra *et al.* 2002; Hazelton *et al.* 2019) [for additional evidence, see Haun and Tononi (2019)].

Several counterintuitive predictions also follow from this account. For example, (i) in cortical areas arranged in a grid-like manner, both active and inactive neurons should support spatial experience; (ii) changes in connectivity in these areas should warp spatial experience even in the absence of changes in activity; (iii) loss of lateral connections should produce a contraction of experienced space of which the subject is unaware (intrinsic scotomas); (iv) grid-like connectivity in occipital areas should support spatial experience devoid of visual qualities in subjects blind from birth (Cattaneo and Vecchi 2011); and (v) changes in the way space feels may be dissociable from changes in performance. The last prediction is illustrated in a companion paper (Grasso *et al.* 2021). By simulating simple neural networks inspired by the organization of cortical and subcortical circuits, we show that visual functions, such as fixating a target, can be performed equivalently by a grid-like substrate (with lateral connections) and by a map-like substrate (organized topographically but lacking lateral connections). Despite their functional equivalence, only the grid specifies a cause–effect structure that can account for spatial experience (Grasso *et al.* 2021).

### Consciousness: what it does or what it is?

We have given an overview of how IIT pursues an objective account of subjective properties, using phenomenal space as a first example. Now let us reconsider the claim that the only way to study consciousness scientifically is by evaluating what we can report about experience—what we can do with it. What would be lost in a purely functional account of space? In a word, everything.

Take again the initial example of the screen and the flashed spots. From a strictly functionalist perspective, as advocated by Cohen and Dennett (2011), all there is to say objectively about the experience is what I can access and report at a given moment. As an experimental subject, I might simply report, “a vast, empty canvas” (1A), or “a dashed spot flashed over a background, slightly to the left” (1B), or “a couple of overlapping spots on the left” (1C), or “a spot including another on the right” (1D), or “five spots at seemingly random locations, which I can point out” (1E), or, finally, “many spots all over the canvas, can’t tell where each was and which overlapped” (1F). The investigators would conclude that this report in fact conveys all I am conscious of. It is all that is accessible to me at any given time as well as all that is amenable to a scientific account.

Indeed, any one of my statements above is certainly adequate to convey what I am experiencing to another human being. Unlike a painting by Jackson Pollock, the experience of a “vast, empty canvas” can be communicated with a message worth just a few bits of Shannon information. But the only reason the report is meaningful is because it can trigger a similar experience in most other human subjects. If they did not experience spatial extendedness in the first place, they would have no idea what I am talking about. The message may be short, but the experience that triggers it and that it evokes is an immense intrinsic structure—one that makes it feel spatial—even though I can only report a few of its components at a time (how could an entire structure be communicated at once?).

Consider distance as an example of this intrinsic structure (Fig. 1E). At any one time, if I am asked to attend to the distance between two dashed spots—say, the two in the top left—I can provide an adequate estimate. But I should also realize that to see any two dashed spots at a certain distance from each other, I must be experiencing all the spots that lie between them (although they are not dashed). Now consider that the image actually contains five dashed spots at seemingly random locations (which I can still report). There are 10 pairwise distances between the five spots as well as distances between each spot and the edges of the screen. I can certainly not report all of those distances, one by one, if the image is flashed briefly. Yet how could I see the spots located where they are if I did not see how far apart they are or how far from the edges of the screen? And how could I see how far apart they are if I did not see all the spots in between? For if those other spots were not there on the canvas, the distance would collapse; all distances would collapse; the very canvas—the feeling of space itself—would collapse.

In fact, even a single spot at the “focus of attention” (e.g. Fig. 1B) already feels extended, like a “region” of space. And how could it feel like a region if it were not composed of many overlapping spots? Furthermore, how could it be experienced as “located” where it is if it were not included by all the other spots that make it feel precisely “there”? Finally, how could the vast canvas that composes the background of that spot feel like a vast canvas if it were not composed of a multitude of spots related among themselves in a particular way? And all those spots and relations, all those regions, locations, and distances are there for me to experience all at once as a vast, extended canvas—an immense spatial structure—even though I can only attend to and report individual spots sequentially, a few at a time.

In summary, there must be a reason why the canvas feels extended rather than, say, like the smell of vanilla. That reason must be the particular way the experience of the canvas is structured. In the case of space, the way experience is structured is at least in part decomposable through introspection—namely, based on the relations of reflexivity, connection, fusion, and inclusion. And once the subjective structure of spatial experience is characterized, albeit partially, we cannot leave it unexplained. Instead, we should try to account for it in objective, physical terms (Haun and Tononi 2019). We should identify a cause–effect structure that corresponds to the phenomenal structure of space in every aspect.

Doing so forces us to evaluate the neural substrate of experience with an eye not on the functions performed by different brain regions, but on the causal structures they support. In this light, it is instructive to revisit standard textbook accounts of spatial vision. The subjective feeling of space and the question of how it can be accounted for in neural terms are nowhere to be found. Instead, the textbooks focus exclusively on how the brain performs spatial functions—how it “represents” external space, operates coordinate transformations, and controls actions such as grasping and pointing. One learns what it takes to correctly grasp an object even though its projection on the retina changes when turning one’s eyes. But one never learns what it takes for the object to be experienced at a particular location in space. Revealingly, in their focus on spatial function rather than experience, the textbooks describe cortical areas in posterior cortex as “maps.” Maps are characterized by a “topographic” correspondence between nearby positions in the cortex and nearby positions in the retina and ultimately in the world. In this view, the neurons that matter are the ones that are activated, and lateral connections are responsible at most for some modulatory effects, although they constitute the bulk of synaptic contacts in posterior areas. At any given moment, inactive neurons do not count, nor do most lateral connections, because they are not “doing anything.” As argued here and elsewhere (Haun and Tononi 2019), however, far from doing nothing, inactive neurons contribute to the cause–effect structure of the system just like active neurons do. And in this way, together with their lateral connections, they are responsible for the experience of space. Accordingly, posterior cortical areas should be thought of as “grids,” not “maps” (Grasso *et al.* 2021).

### Inference to a good explanation: from introspection and reason to neuroscience and back

If we wish to explain consciousness, then, we must account for its intrinsic structure, and to do so we should use all means at our disposal. The starting point must be introspection and reason: without them, we would not even realize there is something to be explained—in fact, we would not even get science started, let alone a science of consciousness. Empirical methods must then be brought in to complement introspection and reason and to extrapolate beyond their reach, in a systematic back-and-forth aiming at a good explanation of phenomenal properties in terms of physical properties.^15^

As argued above, introspective access to one’s private experience—a kind of intrinsic self-report—is the horse that draws the cart of extrinsic report. Introspection and reason are also what allow us to ascribe similar experiences to beings that look and behave like us, and what justify the use of behavioral, functional, and neural correlates in the study of consciousness. Most importantly, introspection and reason allow us to identify properties of experience that must be accounted for in physical terms.

Consider a few examples. As I introspect my experience reading this page, I use reason to infer that I am conscious of the page even when I am not “self-reporting” about it (which is most of the time). To argue that I only see the page when I ask myself about it, or to conclude that either scenario is equally plausible, is nearly as silly as denying the existence of a world independent of my experience or holding that realism and solipsism are equivalent hypotheses. It is silly because realism explains things, while solipsism explains nothing. The same holds for the existence of my experience when I am not self-reporting—its explanatory power makes it the more reasonable conclusion. It is also consistent with empirical evidence: the neural correlates specific to experiencing faces, places, objects, and textures are similar whether we actively report what we see, we view scenes passively (Frässle *et al.* 2014; Tsuchiya *et al.* 2015), or we dream (Siclari *et al.* 2017).

Similarly, it is reasonable to infer that the feeling of extendedness remains largely the same even when I am not self-reporting, that every spot is where it belongs—at its specific location and distance from every other spot—whether I introspect or not. By simple reasoning, I can infer that for a spot to appear where it appears, there must be countless other spots around it that are related to it in specific ways, and for a distance to be experienced as a distance, it must itself be composed of many overlapping spots.

Of course, even at their best, introspection and reason have obvious limitations. For example, attention, working memory, and reasoning (broadly understood as mental manipulation) are largely selective and sequential. At every moment, I can only focus on and hold in mind some aspect of an experience—such as the distance between two spots—and then, say, compare it to another distance. Being able to introspect through attention is critical to access the structure of experience and to reason about it, but at any given moment, my access remains extremely restricted. Introspection is limited in many other ways beyond its selective and sequential features. For example, I cannot penetrate the structure of experienced space down to its smallest components by introspection alone (Haun and Tononi 2019). Furthermore, introspecting the structural properties of other experiences, such as those of a musical chord, is much harder. When it comes to colors and sounds, it seems impossible to introspect any structure at all. And of course, the very act of attending changes what is experienced to some extent. But such limitations simply reflect a truism in science—that every source of evidence has limitations.

Despite their limitations, introspection and reason are the essential tools to bootstrap the scientific study of consciousness—they provide what needs to be explained and highlight its intrinsic structural properties. But then, in order to achieve a good explanation, we must resort to neuroscience. By now we have overwhelming evidence about the strict dependence of experience on the brain. We know that certain neural substrates, but not others, are essential for us to be conscious. We also know that certain brain regions, but not others, are responsible for the specific contents of experience—space, objects, colors, sounds, and so on. We can thus infer that there should be a physical explanation for why certain substrates can support consciousness and others cannot. And there should be a physical explanation for why every experience is structured the way it is.

In the end, a good account of consciousness will require inferences and extrapolations based on a systematic, complementary back-and-forth between introspection, reason, and neuroscientific evidence. Strategically, it seems appropriate to begin with properties of experience that are most amenable to introspection. This is precisely why the feeling of extendedness was chosen for a first attempt at accounting for specific subjective properties in objective, physical terms. First, spatial experiences are pervasive: almost all we see and feel with our body is experienced as spatially extended. Second, and most important, we can at least partially introspect the phenomenal structure of spatial experience: we can attend to spots, their overlaps, and so on, and do so “anywhere” in space. In other words, the intrinsic structure of spatial experience is as “accessible” as we can hope, albeit in a sequential manner due to the limitations of attention, working memory, and executive functions. This accessibility makes it possible to characterize the structure of phenomenal space explicitly, so that we can more easily identify a physical correspondent. Third, in the case of spatial experience, we can suggest a natural candidate as a neural substrate—namely, grid-like areas in posterior cortex—so that the correspondence can be assessed empirically. Finally, there is a likely explanation for the relative accessibility of the intrinsic structure of spatial experiences: our brain can count on powerful mechanisms of spatial attention, which are mediated by changes in excitability conveyed along feedback connections from high-level to low-level visual areas.

The intrinsic structural properties of experienced time and objects can also be partially introspected, although perhaps not as easily and thoroughly as those of space, and ongoing work aims at accounting for them in physical terms. Other contents of experience, however, are much harder to decompose through introspection, even though we realize that they must be structured. For example, take the first chord in Haydn’s “Creation.” I realize that to sound the way it does, the auditory experience I am having is highly structured, but I am simply unable to put that phenomenal structure into words—it is largely ineffable. A potential explanation is that in this case, unlike the case of space, we lack attentional mechanisms that would allow us to use introspection to dissect the distinctions and relations that compose the chord. Or take the black color of the canvas. No matter how hard I try, all I can say is that the color looks imperscrutably black. Its quality is cognitively inaccessible, but it is certainly experienced. For such cases, a scientific approach might start not from introspection but by unfolding the cause–effect structure of neural circuits known to support the experience of colors.

## Conclusion

Much has been gained through a scientific, objective analysis of objective correlates of consciousness. Behavioral correlates are invaluable in everyday life and in the clinic. Functional correlates, based on what we can access and report of our experiences, are necessary for dissecting consciousness and its contents. Neural correlates are critical for discovering the substrate of consciousness in the brain, both of consciousness as a whole and of its various contents.

Many argue that these objective correlates of consciousness are all that can be explained and all that should be explained. Phenomenal properties either do not exist at all, are illusory, or exceed the boundaries of science. Such claims commit the fallacy of misplaced objectivity: they assume that only objective properties should and can be accounted for objectively through science. However, consciousness is about being—not just about doing. While a scientific account of consciousness must be given in objective terms, the object of the account must be its subjective properties. What needs to be explained scientifically is what experience is intrinsically, not just what we can do with it extrinsically. And its properties must be explained; otherwise the way experience feels would turn out to be magical rather than physical.

As briefly summarized here, IIT attempts to account for the intrinsic, subjective properties of consciousness in strictly physical terms—both for the essential properties of every experience and for the specific properties of particular experiences. The tools of IIT provide a way to unfold the cause–effect structure of a physical substrate, such as different brain regions in a particular activity state. The properties of that unfolded substrate can then be examined to establish whether they can account for the essential properties of consciousness. Doing so can explain why certain brain regions support consciousness and others do not, and why they do so in certain activity states and not in others. Furthermore, this approach suggests that certain brain regions, but not others, can support specific phenomenal properties—e.g. that grid-like regions of the brain can support the extendedness of spatial experiences.

According to IIT, even the blackness of black and the painfulness of pain correspond to cause–effect substructures, although we struggle to decompose them from the inside using introspection. But if the central identity of IIT is correct, all quality is structure: all phenomenal properties, including black and pain, are properties of cause–effect structures. Black and pain, too, will have to be accounted for in physical terms—as particular substructures specified by certain cliques of neurons.

Once it is properly appreciated, rather than ignored, the immense structural richness of every human experience—including that of an empty screen—can be a fertile ground for a scientific, objective account. IIT offers a framework that was explicitly developed to pursue this goal. Regardless of the framework, understanding consciousness scientifically means accounting for experience itself—why it is present or absent and why specific experiences feel the way they do. It should be done, and it can be done.^16^

## Data Availability

Data is available upon request.

## Footnotes

1 The reference is to Whitehead’s “fallacy of misplaced concreteness” (Whitehead 1929).

2 One may also note, however, that Watson resorted to subjective criteria, such as seeing, touching, and smelling, to ground the validity of objective criteria.

3 There is now overwhelming empirical evidence that attention can be dissociated from consciousness (Koch and Tsuchiya 2012; Pitts *et al.* 2018).

4 They also rarely consider the evidence in terms of an overall good explanation. Consider an example similar to one introduced later in the main text. When I see a dark screen with, say, three bright dots disposed at random, I see them at their respective position on the screen, and I can prove I do because I can remember their positions and point to them. When I see, say, 30 small bright dots disposed at random, what is the better explanation: (i) that I still see every bright dot at its position, although I cannot remember those positions nor point to them, or (ii) that I suddenly do not see the bright dots at their positions on the screen but only experience a generic feeling of “dotness”? We know there are dedicated, high-level neural mechanisms for remembering a few items but no low-level neural mechanisms for storing the fine details of an image (unlike in a digital camera). We also have good reasons to believe that the neural correlates of the position of each dot can only be found in low-level visual areas, whether the dots are three or thirty. Altogether, it seems that the first hypothesis will turn out to be better than its alternative (Haun And Tononi Submitted).

5 In many cases, sticking with objective correlates of experience may be an instance of substitution bias: “when faced with a difficult question, we often answer an easier one instead, usually without noticing the substitution” (Kahneman 2011).

6 This holds also for the subject of experience: I can only report to myself a few aspects of the experience I am having, due to the limitations of introspection (attention, working memory, and mental manipulation work in a selective, sequential manner). This does not mean, however, that what I experience at any one moment is only what I can report to myself sequentially. I can infer that, for the experience of an extended canvas to feel the way it does, it must be composed of many spots bound by countless relations (see below). In short, phenomenal structure vastly exceeds self-report (or any other report).

To understand this point, see the video tutorial linked in footnote 14.

8 Note that “unfolding” in IIT should not be confused with the use of this term in computer science.

9 IIT’s identity is not meant to reduce the phenomenal (subjective) to the physical (objective) but to emphasize the one-to-one correspondence between the two structures necessary to carry out the explanatory work. If IIT is correct, the cause–effect structure specified by a physical substrate accounts for the experience, as it leaves nothing within the experience unaccounted for.

10 The value of integrated information *Φ* of a system in its current state quantifies how integrated (irreducible) its unfolded cause–effect structure is. To be a physical substrate of consciousness, the system must be a maximum of integrated information *Φ*, across its constituents, their possible states, and spatiotemporal scales.

11 These studies, it is worth noting, systematically employ behavioral, functional, and neural correlates of consciousness. For example, subjects may be instructed to report whether they had experienced anything just before they are awakened from sleep. Sometimes they report having dreamt, and sometimes they report “emerging from nothingness.” Brain activity a few seconds before awakening can then be used to assess what brain areas and what kinds of neural activity are correlated systematically with the presence or absence of consciousness, as assessed by the subjects’ delayed reports. Subjects often report that they had been dreaming just before awakening but they cannot remember the content of the dream. Neural correlates can also be exploited to test the hypothesis that during such “white dreams,” the same areas were activated as in remembered dreams, but brain regions important for storing or retrieving episodic memories were deactivated (Siclari *et al.* 2017). Behavioral, functional, and neural correlates can thus be deployed together to arrive at a satisfactory account of the experimental results. Most importantly, they can be used to validate theoretical predictions about the physical requirements that must be satisfied to account for the essential properties of consciousness. Indeed, the posterior cortical areas that emerge as the main neural correlates of dreaming sleep are organized anatomically in a way that seems ideally suited to support high values of *Φ*. Furthermore, the main difference between dreaming and dreamless sleep is the occurrence of slow waves in these posterior areas. And sleep slow waves are an expression of neuronal bistability that disrupts causal interactions and the integration of information (Pigorini *et al.* 2015; Tononi *et al.* 2016). As stated in the main text, a good explanation of consciousness calls for inferences based on all the tools at our disposal, from introspection to extrinsic reports to neuroscientific evidence.

12 Note that the feeling of extendedness would be there irrespective of whether the canvas is black, white, or blue; whether it is empty or full of objects; or whether it is an imagined canvas, an experimental “Ganzfeld,” or a real-world canvas of the sky or the ocean. For present purposes, we ignore the fact that my current experience also feels “black” and that I have in mind the concepts “vast,” “empty,” and “canvas.”

13 In this context, it is ironic that Descartes and others considered extendedness the defining property of matter—the *res extensa*—in contrast to mind—the *res cogitans*—which was not extended.

14 For a brief video tutorial about the fundamental phenomenal properties of spatial experience, see https://centerforsleepandconsciousness.psychiatry.wisc.edu/index.php/space-tutorial.

15 In other words, in accounting for consciousness—as in every other scientific pursuit—we must use inference to a good explanation. We prefer to say “good” rather than the more common “best” (Lipton 1991) because in science, as in life, it is often hard to know what is best, but it may be possible to know what is good enough, at least for the moment. In general, a good explanation should (i) account for many facts (“scope”), (ii) explain them precisely (“specificity”), (iii) be internally coherent (“self-consistency”), (iv) be coherent with our overall view of things (“system consistency”), (v) be simpler than alternatives (“simplicity”), and (vi) make testable predictions (“scientific validation”).

16 The reference is to Hilbert’s response to du Bois-Reymond’s “ignoramus et ignorabimus” (we do not know and will not know): “Wir müssen wissen—wir werden wissen” (We must know—we will know).

## References

[R1] Balduzzi D , Tononi G. Integrated information in discrete dynamical systems: motivation and theoretical framework. *PLoS Comput Biol* 2008;4:e1000091. 10.1371/journal.pcbi.1000091 PMC2386970 18551165

[R2] Bisiach E , Luzzatti C. Unilateral neglect of representational space. *Cortex* 1978;14:129–33. 16295118 10.1016/s0010-9452(78)80016-1

[R3] Block N . Perceptual consciousness overflows cognitive access. *Trends Cogn Sci* 2011;15:567–75. 22078929 10.1016/j.tics.2011.11.001

[R4] Block N . Rich conscious perception outside focal attention. *Trends Cogn Sci* 2014;18:445–7. 24890010 10.1016/j.tics.2014.05.007

[R5] Bruno MA , Vanhaudenhuyse A, Schnakers C et al. Visual fixation in the vegetative state: an observational case series pet study. *BMC Neurol* 2010;10:35. 10.1186/1471-2377-10-35 PMC2895583 20504324

[R6] Butter CM , Kosslyn S, Mijovic-Prelec D et al. Field-specific deficits in visual imagery following hemianopia due to unilateral occipital infarcts. *Brain* 1997;120:217–28. 9117370 10.1093/brain/120.2.217

[R7] Casali AG , Gosseries O, Rosanova M et al. A theoretically based index of consciousness independent of sensory processing and behavior. *Sci Transl Med* 2013;5:198ra105. 10.1126/scitranslmed.3006294 23946194

[R8] Casarotto S , Comanducci A, Rosanova M et al. Stratification of unresponsive patients by an independently validated index of brain complexity. *Ann Neurol* 2016;80:718–29. 27717082 10.1002/ana.24779 PMC5132045

[R9] Cattaneo Z , Vecchi T. *Blind Vision: The Neuroscience of Visual Impairment*. Cambridge: MIT Press, 2011.

[R10] Chalmers DJ . What is a neural correlate of consciousness? In: Metzinger T (ed.), *Neural Correlates of Consciousness: Empirical and Conceptual Questions*. Oxford: The MIT Press, 2000, 17–39.

[R11] Churchland PM . Eliminative materialism and propositional attitudes. *J Philos* 1981;78:67–90.

[R12] Cohen MA , Dennett DC. Consciousness cannot be separated from function. *Trends Cogn Sci* 2011;15:358–64. 21807333 10.1016/j.tics.2011.06.008

[R0014a] Crick F , Koch C. Towards a neurobiological theory of consciousness. *InSeminars in the Neurosciences*, Vol. 2. Saunders Scientific Publications, 1990, 263–75.

[R13] Crick F , Koch C. A framework for consciousness. *Nat Neurosci* 2003;6:119–26. 12555104 10.1038/nn0203-119

[R14] Davis T , Poldrack RA. Measuring neural representations with fMRI: practices and pitfalls. *Ann N Y Acad Sci* 2013;1296:108–34. 23738883 10.1111/nyas.12156

[R15] Diedrichsen J , Kriegeskorte N. Representational models: a common framework for understanding encoding, pattern-component, and representational-similarity analysis. *PLoS Comput Biol* 2017;13:e1005508. 10.1371/journal.pcbi.1005508 PMC5421820 28437426

[R16] Doerig A , Schurger A, Hess K et al. The unfolding argument: why IIT and other causal structure theories cannot explain consciousness. *Conscious Cogn* 2019;72:49–59. 31078047 10.1016/j.concog.2019.04.002

[R17] Farah MJ , Soso MJ, Dasheiff RM. Visual angle of the mind’s eye before and after unilateral occipital lobectomy. *J Exp Psychol Hum Percept Perform* 1992;18:241–6. 1532190 10.1037//0096-1523.18.1.241

[R18] Fechner GT . Elements of psychophysics, 1860. In: Dennis W. (ed.), Readings in the history of psychology. Appleton-Century-Crofts, 1948, 206–13.

[R19] Frankish K . Illusionism as a theory of consciousness. *J Conscious Stud* 2016;23:11–39.

[R20] Frankish K . Panpsychism and the depsychologization of consciousness. *Aristotelian Soc Suppl Volume* 2021;95:51–70.

[R21] Frässle S , Sommer J, Jansen A et al. Binocular rivalry: frontal activity relates to introspection and action but not to perception. *J Neurosci* 2014;34:1738–47. 24478356 10.1523/JNEUROSCI.4403-13.2014 PMC6827584

[R22] Fu Y , Yan W, Shen M et al. Does consciousness overflow cognitive access? Novel insights from the new phenomenon of attribute amnesia. *Sci China Life Sci* 2021;64:847–60. 33515433 10.1007/s11427-020-1831-8

[R23] Gazzaniga MS , Ivry RB, Mangun GR et al. *Cognitive Neuroscience: The Biology of the Mind* . New York: W. W.Norton & Company, 2019.

[R24] Gilhotra JS , Mitchell P, Healey PR et al. Homonymous visual field defects and stroke in an older population. *Stroke* 2002;33:2417–20. 12364731 10.1161/01.str.0000037647.10414.d2

[R25] Grasso M , Haun A, Tononi G. Of maps and grids. Neurosci conscious 2021;7:1–10. 10.1093/nc/niab022 PMC8452603 34557311

[R26] Graziano MS . *Rethinking Consciousness: A Scientific Theory of Subjective Experience*. New York: WW Norton & Company, 2019.

[R27] Haun A , Tononi G. Why does space feel the way it does? Towards a principled account of spatial experience. *Entropy* 2019;21:1160.

[R28] Haun A , Tononi G. (Forthcoming) Do you see all the dots?

[R29] Haun AM , Tononi G, Koch C et al. Are we underestimating the richness of visual experience? *Neurosci Conscious* 2017;3. 10.1093/nc/niw023 PMC6007133 30042833

[R30] Haynes J-D , Rees G. Predicting the orientation of invisible stimuli from activity in human primary visual cortex. *Nat Neurosci* 2005;8:686–91. 15852013 10.1038/nn1445

[R31] Hazelton C , Pollock A, Taylor A et al. A qualitative exploration of the effect of visual field loss on daily life in home-dwelling stroke survivors. *Clin Rehabil* 2019;33:1264–73. 30935223 10.1177/0269215519837580

[R32] Horikawa T , Tamaki M, Miyawaki Y et al. Neural decoding of visual imagery during sleep. *Science* 2013;340:639–42. 23558170 10.1126/science.1234330

[R33] Kahneman D . *Thinking, Fast and Slow*. Farrar: Straus and Giroux, 2011.

[R34] Koch C , Massimini M, Boly M et al. Neural correlates of consciousness: progress and problems. *Nat Rev Neurosci* 2016;17:307–21. 27094080 10.1038/nrn.2016.22

[R35] Koch C , Tsuchiya N. Attention and consciousness: related yet different. *Trends Cogn Sci* 2012;16:103–5. 22154091 10.1016/j.tics.2011.11.012

[R36] Kriegeskorte N , Douglas PK. Interpreting encoding and decoding models. *Curr Opin Neurobiol* 2019;55:167–79. 31039527 10.1016/j.conb.2019.04.002 PMC6705607

[R37] Linassi F , Zanatta P, Tellaroli P et al. Isolated forearm technique: a meta-analysis of connected consciousness during different general anaesthesia regimens. *Br J Anaesth* 2018;121:198–209. 29935574 10.1016/j.bja.2018.02.019

[R38] Lipton P . *Inference to the Best Explanation*. New York: Routledge, 1991.

[R39] Massimini M , Ferrarelli F, Huber R et al. Breakdown of cortical effective connectivity during sleep. *Science* 2005;309:2228–32. 16195466 10.1126/science.1117256

[R40] Oizumi M , Albantakis L, Tononi G. From the phenomenology to the mechanisms of consciousness: integrated information theory 3.0. *PLoS Comput Biol* 2014;10:e1003588. 10.1371/journal.pcbi.1003588 PMC4014402 24811198

[R41] Owen AM , Coleman MR, Boly M et al. Detecting awareness in the vegetative state. *Science* 2006;313:1402. 10.1126/science.1130197 16959998

[R42] Pigorini A , Sarasso S, Proserpio P et al. Bistability breaks-off deterministic responses to intracortical stimulation during non-rem sleep. *NeuroImage* 2015;112:105–13. 25747918 10.1016/j.neuroimage.2015.02.056

[R43] Pitts MA , Lutsyshyna LA, Hillyard SA. The relationship between attention and consciousness: an expanded taxonomy and implications for ‘no-report’ paradigms. *Philos Trans R Soc B-Biol Sci* 2018;373:20170348. 10.1098/rstb.2017.0348 PMC6074089 30061462

[R44] Poldrack RA . Inferring mental states from neuroimaging data: from reverse inference to large-scale decoding. *Neuron* 2011;72:692–7. 22153367 10.1016/j.neuron.2011.11.001 PMC3240863

[R45] Pollen DA . On the neural correlates of visual perception. *Cereb Cortex* 1999;9:4–19. 10022491 10.1093/cercor/9.1.4

[R46] Posner JB , Saper CB, Schiff ND et al. *Plum and Posner’s Diagnosis and Treatment of Stupor and Coma* . 5th edn. Oxford: Oxford University Press, 2019.

[R47] Postle BR . *Essentials of Cognitive Neuroscience*. 2nd edn. Hoboken: Wiley, 2020.

[R48] Sanders RD , Tononi G, Laureys S et al. Unresponsiveness ≠ unconsciousness. *Anesthesiology* 2012;116:946–59. 22314293 10.1097/ALN.0b013e318249d0a7 PMC3311716

[R49] Sarasso S , Boly M, Napolitani M et al. Consciousness and complexity during unresponsiveness induced by propofol, xenon, and ketamine. *Curr Biol* 2015;25:3099–105. 26752078 10.1016/j.cub.2015.10.014

[R50] Siclari F , Baird B, Perogamvros L et al. The neural correlates of dreaming. *Nat Neurosci* 2017;20:872–8. 28394322 10.1038/nn.4545 PMC5462120

[R51] Sperry RW . Hemisphere deconnection and unity in conscious awareness. *Am Psychol* 1968;23:723–33. 5682831 10.1037/h0026839

[R52] Tononi G . Integrated information theory. *Scholarpedia* 2015;10:4164.

[R53] Tononi G , Boly M, Massimini M et al. Integrated information theory: from consciousness to its physical substrate. *Nat Rev Neurosci* 2016;17:450–61. 27225071 10.1038/nrn.2016.44

[R54] Tsuchiya N , Wilke M, Frassle S et al. No-report paradigms: extracting the true neural correlates of consciousness. *Trends Cogn Sci* 2015;19:757–70. 26585549 10.1016/j.tics.2015.10.002

[R55] Usher M , Bronfman ZZ, Talmor S et al. Consciousness without report: insights from summary statistics and inattention ‘blindness’. *Philos Trans R Soc B-Biol Sci* 2018;373:20170354. 10.1098/rstb.2017.0354 PMC6074079 30061467

[R56] Watson JB , McDougall W. *The Battle of Behaviorism: An Exposition and an Exposure*. W.W. Norton & Co, 1929.

[R57] Whitehead AN . *Process and Reality: An Essay in Cosmology*. New York: The Macmillan, 1929.

[R58] Wundt WM . *Principles of Physiological Psychology*. London: Sonnenschein, 1904.

